# Real-World Evidence in Drug Approvals at the European Medicines Agency

**DOI:** 10.1001/jamanetworkopen.2025.42041

**Published:** 2025-11-06

**Authors:** Magdalena Bachinger, Maciej A. Jankowski, Aaron S. Kesselheim, Nils Krüger

**Affiliations:** 1 Center for Digital Technology Management, Technical University Munich, Munich, Germany; 2 Department of Cardiovascular Diseases, German Heart Center, TUM University Hospital, Technical University Munich, Munich, Germany; 3 Program On Regulation, Therapeutics, And Law (PORTAL), Division of Pharmacoepidemiology and Pharmacoeconomics, Department of Medicine, Brigham and Women’s Hospital/Harvard Medical School, Boston, Massachusetts; 4 Division of Pharmacoepidemiology and Pharmacoeconomics, Department of Medicine, Brigham and Women’s Hospital/Harvard Medical School, Boston, Massachusetts

## Abstract

This cross-sectional study assesses how the European Medicines Agency incorporated routinely collected clinical data into its approval processes from 2020 to 2023.

## Introduction

Integrating evidence generated during routine clinical use (real-world evidence) into regulatory science is important for addressing gaps left by randomized clinical trials, which often do not reflect clinical practice. In recent years, real-world evidence gained wide attention, particularly in the US, playing a growing role in regulatory decision-making by the US Food and Drug Administration (FDA). Thirty-one percent of FDA approvals from 2019 to 2021 incorporated data generated during routine clinical use, as reported by Purpura et al.^1^ While FDA and European Medicines Agency (EMA) decisions are highly concordant,^2^ little is known about how the EMA, as the second-largest regulator, incorporates such data into its approval processes.

## Methods

This cross-sectional study used publicly available EMA assessment reports, did not include human participants, and was determined exempt from ethical review and the requirement for informed consent per US regulation (45 CFR 46) and EU regulation 2016/679 recital 26. The study followed the STROBE reporting guideline. We systematically analyzed EMA assessment reports for real-world evidence from 2020 to 2023, which reflect the agency’s evaluation of submitted applications (eMethods in Supplement 1). All eligibility criteria, keyword terms, and extracted variables are summarized in the Figure. First, assessment reports were selected according to predefined inclusion criteria. Second, a targeted keyword search was performed and screened against inclusion criteria. Third, 17 variables were extracted from each study in the final cohort (eMethods in Supplement 1). Steps 2 and 3 were completed independently by 2 researchers (M.B., M.A.J.) to ensure reliability, with final validation by a senior researcher (N.K.) and any disagreement resolved by consensus. Data were analyzed using Excel (Microsoft).

**Figure.**
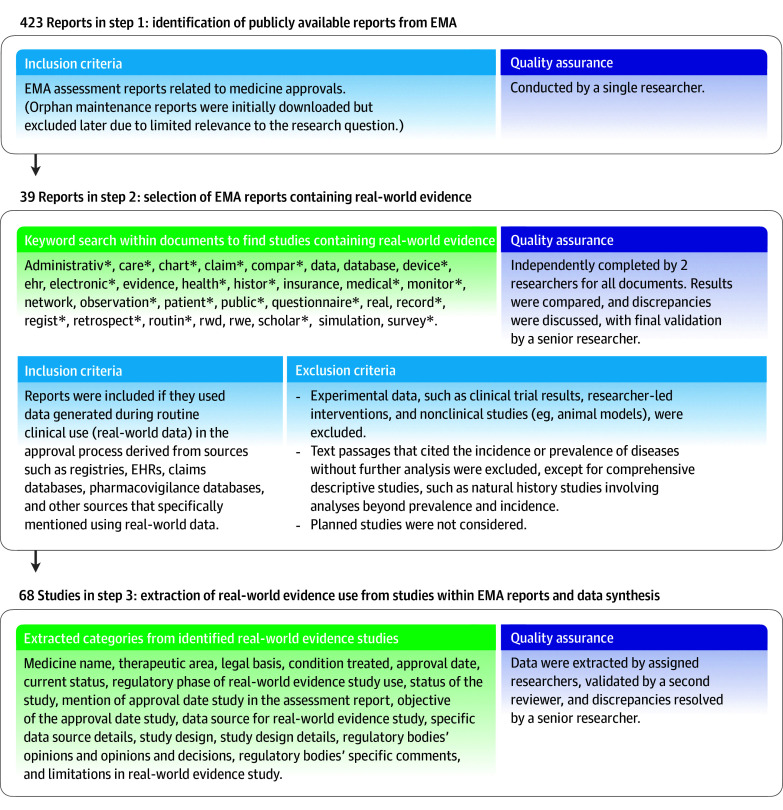
Eligibility Criteria, Keyword Terms, and Extracted Variables From Reports Related to Medicine Approvals by the EMA Between 2020 and 2023 In step 1, the researcher is M.B. In step 2, the 2 researchers are M.B. and M.A.J. The senior researcher is N.K. In step 3, the assigned researchers were M.B. and M.A.J.; validation was performed by the researcher who was not assigned to each item (M.B. validated the work of M.A.J. and vice versa), and discrepancies were resolved by N.K. EHRs indicates electronic health records; EMA, European Medicines Agency.

## Results

Of the 423 identified reports, 39 (9.2%) incorporated real-world evidence, encompassing 68 relevant studies. Twenty studies (29.4%) were from reports issued in 2020, 24 (35.3%) from 2021, 13 (19.1%) from 2022, and 11 (16.1%) from 2023. Among all investigated applications, 36 (92.3%) remain authorized by June 1, 2025. Two of the 3 withdrawn applications related to COVID-19.

Twenty studies were excluded from data analysis because the information provided could not be verified or was incomplete (11 cases). Of the remaining 48, most used a cohort design, with objectives including descriptive analyses, safety, and benefit evaluation. The data sources included registries (22 [45.8%]), electronic health records (16 [33.3%]), claims databases (3 [6.3%]), and pharmacovigilance databases (1 [2.1%]), while 11 (22.9%) did not specify data sources. Oncology (18 [37.5%]) was the predominant therapeutic area, followed by COVID-19 (7 [14.6%]) and neurology (6 [12.5%]) (Table). Explicit regulatory endorsement for real-world evidence studies remained limited, appearing along with only 14 (29.2%) studies (Table).

**Table.** Overview of Real-World Evidence Studies in EMA Reports Related to Medicine Approvals

Category of analysis^a^	No. (%)
Assessment reports including real-world evidence studies in the approval process, No.	39
Studies including real-world evidence, No.	
Total	68
Included in this study	48/68 (70.1)
Excluded due to lack of information	20/68 (29.4)
Real-world evidence studies per assessment report, No.	
1	34
≥2	5
Current status of pharmaceutical	
Authorized	40/48 (83.3)
Withdrawn	8/48 (16.7)
Status of the study	
Completed	40/48 (83.3)
Ongoing	8/48 (16.7)
Approval date	
2020	11/48 (22.9)
2021	22/48 (45.8)
2022	11/48 (22.9)
2023	4/48 (8.3)
Therapeutic area	
Cancer	18/48 (37.5)
Cardiovascular	1/48 (2.1)
COVID-19	7/48 (14.6)
Endocrinology	3/48 (6.3)
Gastroenterology/hepatology	1/48 (2.1)
Hematology/hemostaseology	2/48 (4.2)
Immunology/rheumatology/transplantation	1/48 (2.1)
Infections	1/48 (2.1)
Metabolism	4/48 (8.3)
Neurology	6/48 (12.5)
Pneumology/allergology	3/48 (6.3)
Reproductive	1/48 (2.1)
Objective of the real-world evidence study^b^	
Safety	18/48 (37.5)
Efficacy/effectiveness	34/48 (70.1)
Descriptive	4/48 (8.3)
Type of real-world data source^b^	
EHR database	16/48 (33.3)
Registry	22/48 (45.8)
Pharmacovigilance database	1/48 (2.1)
Health care claims database	3/48 (6.3)
Not mentioned	11/48 (22.9)
Study design	
Cohort study	42/48 (87.5)
Descriptive study	2/48 (4.2)
Not categorizable	4/48 (8.3)
Regulatory bodies’ opinions/decisions	
Not supporting use of real-world data/evidence	7/48 (15.6)
Supporting use of real-world data/evidence	14/48 (29.2)
Addressed but not categorizable	4/48 (8.3)
Unable to determine origin	3/48 (6.3)
Not addressed	20/48 (41.7)
Regulatory characteristics^b^	
New active substance	33/39 (84.6)
PRIME^c^	9/39 (23.1)
Orphan medicine	17/39 (43.6)
Accelerated assessment	2/39 (5.1)
Conditional marketing authorization	18/39 (46.2)
ATMP^d^	6/39 (15.4)
Approval under exceptional circumstances	4/39 (10.3)
No information given	2/39 (5.1)

Abbreviations: ATMP, advanced therapy medicinal products; EMA, European Medicines Agency; PRIME, priority medicines.

^a^ A detailed description of all variables and raw data are available in the eMethods in Supplement 1.

^b^ Percentages do not add up to 100% because some studies may assign multiple levels of the variable to one included study.

^c^ PRIME is an EMA scheme that supports the development of medicines addressing unmet medical needs.

^d^ ATMPs are gene-based, tissue-based, or cell-based medicines that offer innovative treatment options for disease and injury.

## Discussion

In this study, fewer than 1 in 10 approvals from 2020 to 2023 incorporated real-world evidence, showing its modest role in EMA decision-making at first marketing authorization.

We observed a lack of uniformity in how real-world evidence is defined and presented across EMA committees. Public assessment reports rarely explained how these studies affected benefit-risk conclusions. This limited transparency may leave applicants uncertain about expectations and dampen incentives to submit real-world evidence.

The absence of EMA-specific guidance on reporting real-world evidence may have contributed to inconsistent assessment reports; many lacked clear structure, stated study objectives vaguely, and used the terms *efficacy* and *effectiveness* interchangeably. Adopting a dedicated standardized submission template, such as the Harmonized Protocol Template endorsed by the FDA and Medicare, could improve transparency, reproducibility, and confidence in real-world evidence submissions.^3^ This study is limited by reliance on public EMA reports, which may not reflect all regulatory considerations of real-world evidence.

Momentum is building across Europe for the integration of high-quality real-world evidence into drug regulation. The EU Data Analysis and Real World Interrogation Network initiative is creating a robust data infrastructure, with increasing real-world evidence adoption in recent years.^4^ Also, recent legislative changes in Germany and France have expanded access to national health care databases, increasing the availability of routine clinical data for research.^5,6^ We thus expect to see an upward trend in the regulatory use of real-world evidence in future years at EMA.

## Notes

Supplement 1. **eMethods.** Detailed Overview of Study Methodology

Supplement 2.
Data Sharing Statement
