# Supplementary material for: Real-World Evidence in Drug Approvals at the European Medicines Agency
Source: JAMA Netw Open. 2025 Nov 6;8(11):e2542041. doi: 10.1001/jamanetworkopen.2025.42041 (PMC12593103; doi:10.1001/jamanetworkopen.2025.42041)
Supplement: Supplement 1. — eMethods. Detailed Overview of Study Methodology [file jamanetwopen-e2542041-s001.pdf]

Supplemental Online Content

Bachinger M, Jankowski MA, Kesselheim AS, Krüger N. Routine clinical evidence in drug approvals at the European Medicines Agency. *JAMA Netw Open.* 2025;8(11):e2542041. doi:10.1001/jamanetworkopen.2025.42041

eMethods. Detailed Overview of Study Methodology

This supplemental material has been provided by the authors to give readers additional information about their work.

© 2025 Bachinger M et al. *JAMA Network Open*.

eMethods Detailed Overview of Study Methodology

The following provides a comprehensive overview and detailed description of the study methodology's development and application.

To address the research question, *"To what extent was real-world evidence (RWE) integrated into European Medicines Agency (EMA) regulatory assessments of newly marketed medicines between 2020 and 2023, and what does this reveal about its role in European drug approvals?"*, we developed a three-step research approach to assess reports on newly marketed medicines within the European Union. Through iterative testing, we ensured alignment with the research question. We conducted pilot runs that identified areas for methodological improvement, leading to targeted revisions for improved study rigor and clarity.

To promote transparency, a complete overview of all analytical steps and associated data is publicly available online under the following link: https://drive.google.com/drive/folders/1t-WnhXkc4fL41qRmV2mgVL04gKvbKk9x?usp=sharing

© 2025 Bachinger M et al. *JAMA Network Open*.

Step 1: Identification of publicly available reports from EMA: [1]

The European Medicines Agency (EMA) publishes annual highlights that provide a comprehensive summary of its activities each year. These include marketing authorization and post-marketing surveillance activities for both human and veterinary medicines.[2] Reports related to human medicines from 2020 to 2023 were obtained to serve as a foundational resource for identifying and analyzing medicines relevant to this study. Each listed medicine was further examined through the EMA's medicine database.[3] Searching for a medicine in the database typically yielded multiple results. This study considered EMA assessment reports and orphan maintenance reports essential to be included in the analysis. To ensure completeness and transparency, we also provided a list of the document types that were excluded. This list reflects the intended purpose of the documents rather than their exact titles, as wording varied across documents.

- Withdrawal reports
- Meeting minutes
- Q&A reports
- Summary of opinion(s)
- Risk-management-plan summary
- All authorized presentations
- Medicine overview
- Product information
- Procedural steps taken and scientific information after authorization
- Paediatric investigation plan compliance statement
- Orphan designation withdrawal assessment report
- Conditions imposed on member states for safe and effective use
- Withdrawal letter
- Refusal public assessment report
- Acceptance of a modification of an agreed paediatric investigation plan
- Public statement
- Periodic safety update report
- Scientific conclusions and grounds for the variation to the terms
- Timetable for procedures
- Direct healthcare professional communication
- Supply shortage reports

To ensure consistency and reproducibility of results, the publication date in the reports was used to determine the year of inclusion, as these dates sometimes differ from those listed in the EMA database for approved medicines[3]. Reports of medicines initially approved but later withdrawn were included, as this study focuses on the initial approval phase rather than subsequent withdrawals.
This initial step was conducted by a single researcher (Magdalena Bachinger) and resulted in the identification of 423 relevant documents. All selected documents are accessible in the folder "03_Selected Documents"[1].

[1] Full documentation of the methodology (see 01_Study Methods) and data: https://drive.google.com/drive/folders/1t-WnhXkc4fL41qRmV2mgVL04gKvbKk9x?usp=sharing

[2] Annual medicine highlights: https://www.ema.europa.eu/en/about-us/what-we-do/authorisation-medicines/medicine-evaluation-figures#annual-medicines-highlights-649533

[3] EMA database of medicines: https://www.ema.europa.eu/en/medicines

© 2025 Bachinger M et al. *JAMA Network Open*.

Step 2: Selection of EMA reports containing RWE:

Step 2 involved creating a spreadsheet[4] to list all documents selected in the previous step. Two researchers independently screened these documents for relevant keywords(see list below) and assessed their context to determine whether the approval process for these medicines included real-world data (RWD) or real-world evidence. According to the EMA, "RWD are data that describe patient characteristics (including treatment utilization and outcomes) in routine clinical practice", and "RWE is evidence derived from the analysis of RWD" (European Medicines Agency, 2024). Each researcher, working without knowledge of the other's assessments, recorded decisions directly in the spreadsheet, providing brief justifications for inclusion. The assessments are stored in separate tabs labeled "Step1_Researcher1" (Magdalena Bachinger) and "Step1_Researcher2" (Maciej Jankowskit[4]).

The following keywords were applied to identify relevant passages within the documents:

1. *administrativ**
2. *care**
3. *chart**
4. *claim**
5. *compar**
6. *data*
7. *database*
8. *device**
9. *ehr*
10. *electronic**
11. *evidence*
12. *health**
13. *histor**
14. *insurance*
15. *medical**
16. *monitor**
17. *network*
18. *obeservation**
19. *patient**
20. *public**
21. *questionnaire**
22. *real*
23. *record**
24. *regist**
25. *retrospect**
26. *routin**
27. *rwd*
28. *rwe*
29. *scholar**
30. *simulation*
31. *survey**

[4] See 04_Data Extraction: https://drive.google.com/drive/folders/1t-WnhXkc4fL41qRmV2mgVL04gKvbKk9x?usp=sharing

© 2025 Bachinger M et al. *JAMA Network Open*.

Inclusion Criteria:

To determine whether a document should be included, the reports had to utilize RWD in the approval process, derived from sources such as registries, electronic health records (EHR), claims databases, pharmacovigilance databases, and other sources that specifically mentioned making a use of RWD.

Exclusion Criteria:

Documents were excluded if they contained experimental data, such as clinical trial results, researcher-led interventions, and non-clinical studies (e.g., animal models). Another criterion for an exclusion were text passages in the reports that merely cited the incidence prevalence of diseases without further analysis were excluded, except for comprehensive descriptive studies, such as natural history studies involving analyses beyond prevalence and incidence. Planned studies were also not considered.

The senior researcher (Dr. med. Nils Krüger) then reviewed the results in the “Step1_SeniorResearcher” tab. In cases where discrepancies arose between the two assessments, the senior researcher conducted a re-evaluation together with the two researchers to make a final decision on inclusion.

© 2025 Bachinger M et al. *JAMA Network Open*.

Step 3: Extraction of RWE use from studies within EMA reports and data synthesis: For this step ("Step2" tab)[4], the reports identified in the previous steps were split into two sets and assigned to the researchers. The guidelines followed throughout this process, which are outlined below, guaranteed consistency and accuracy for its analysis:

- Each researcher extracted data from their assigned assessment reports based on 17 predefined columns.
- When multiple relevant RWE studies were identified within a single assessment report, a new row was created for each study.
- If the information was implied but not explicitly stated, it was marked respectively ([AS] for assumption).
- Statements not quoted verbatim but summarized or modified were shaded in gray for transparent reporting.
- Assessment reports with reference document names ending with "O" - representing the orphan maintenance assessment reports mentioned in Step 1 - were excluded at this stage. Initially, these reports were considered relevant based on the assumption that orphan maintenance documents might include information on RWE usage. However, upon thorough review, it became evident that those documents rarely provided information about the approval process. Consequently, they were excluded during Step 3.

The following columns were used for data extraction. The list below also includes brief explanations of each column:

- **Study ID:** Assigned to simplify the creation of descriptive statistics in later steps.
- **Step1: Include/Exclude:** Taken from tab Step1_SeniorResearcher, Column SeniorResearcher: Include/Exclude; Indication from the reviewer if the report should be included, excluded or considered.
- **Step2: Include/Exclude:** Studies that were included or considered from Step1: Include/Exclude were thoroughly checked, and it was indicated whether they should be included, excluded because the initial inclusion criteria were not met (not covered by the inclusion criteria), or excluded due to a lack of information to further progress with the study at this step.
- **Reference Document:** Reports downloaded from the EMA database; Naming convention: Year_Medicine Name_Ending of Document Optional: Added number; Ending of Document: AR = Assessment Report of the General Assessment, O = Assessment Report of the Orphan Assessment, Added number: When there was more than one AR or O report per medicine, additional numbering was used, e.g., AR2 for a second assessment report.

© 2025 Bachinger M et al. *JAMA Network Open*.

Documents can be found in the folder "03_Selected Documents" in the aforementioned Google Drive.

- **Medicine Name:** Taken from the EMA database.[3]
- **Therapeutic Area:** Taken from EMA's published Medicine Overview documents.[2]
- **Regulatory Characteristics:** Taken from EMA's published Medicine Overview documents.[2]
- **Condition Treated:** Taken from the report (abbreviated).
- **Approval Date:** Issuance year of the assessment report: Since the EMA database and the reports occasionally had differing dates, the year stated within the report was utilized.
- **Current Status of Pharmaceutical:** Current authorization status of the medicine within Europe (authorized or withdrawn) taken from the EMA database.[3]
- **Regulatory Phase of RWE Study Utilization**: Indicates whether the respective study was/ will be utilized during the pre-authorization phase (before marketing authorization) or the post-authorization phase (after marketing authorization).
- **Status of the Study:** Indication taken from the report whether the respective study is ongoing or completed.
- **Mention of RWE Study in the AR:** Chapter or context in which the respective study was presented in the assessment report.
- **Objective of the RWE Study:** Objective/Goal for which the RWE study was conducted (Descriptive, Safety, Effectiveness/ Efficacy).
- **Type of RWD Source:** Data utilized for the study (Registry, EHR database, Claims database, Pharmacovigilance database, Not mentioned).
- **Specific Data Source Details:** Further details mentioned in the report regarding the data source.
- **Study Design:** Study design used for the respective study (Cohort study, Case-control study, Cross-sectional study, Descriptive study, Not categorizable). The "Not categorizable" category includes studies whose reports lack sufficient design details, and for which even a web search did not clarify the exact study design. To help classify the studies, a research paper published by Taur, 2022 was used.
- **Study Design Details:** Further details mentioned in the report regarding the data source.
- **Regulatory Bodies' Opinions/Decisions:** The categorization included the following: "Supporting use of RWD/E", indicating clear support from regulatory bodies; "Not supporting use of RWD/E", indicating clear opposition from regulatory bodies; "Addressed but not categorizable", meaning it was unclear

whether the stance was supportive or not; "Unable to determine origin", indicating uncertainty about whether the comment originated from the applicant or regulatory bodies; and "Not addressed" indicating no mention of RWD/E in the document.

- **Regulatory Bodies' Specific Comments:** Comments from regulatory bodies on the RWE study.
- **Limitations in RWE Study:** Limitations noted in the assessment report concerning the RWE study.

The extracted data underwent multiple feedback rounds and discussions, including sessions with the senior researcher, to ensure its accuracy and completeness. The finalized dataset served as the foundation for subsequent analysis and interpretation.

Bibliography

European Medicines Agency. (2024). Reflection paper on the use of real-world data in non-interventional studies to generate real-world evidence. https://www.ema.europa.eu/en/documents/scientific-guideline/draft-reflection-paper-use-real-world-data-non-interventional-studies-generate-real-world-evidence_en.pdf

Taur, S. R. (2022). Observational designs for real-world evidence studies. *Perspectives in Clinical Research*, *13*(1), 12–16. https://doi.org/10.4103/picr.picr_217_21

© 2025 Bachinger M et al. *JAMA Network Open*.
