# Supplementary material for: Real-World Evidence in Drug Approvals at the European Medicines Agency
Source: JAMA Netw Open. 2025 Nov 6;8(11):e2542041. doi: 10.1001/jamanetworkopen.2025.42041 (PMC12593103; doi:10.1001/jamanetworkopen.2025.42041)
Supplement: Supplement 2. — Data Sharing Statement [file jamanetwopen-e2542041-s002.pdf]

Data Sharing Statement

Bachinger. Routine Clinical Evidence in Drug Approvals at the European Medicines Agency. *JAMA Netw Open*. Published November 06, 2025. doi:10.1001/jamanetworkopen.2025.42041

Data
Data available: Yes
Data types: Data (not involving human participants)
How to access data: https://drive.google.com/drive/folders/1t-WnhXkc4fL41qRmV2mgVL04gKvbKk9x?usp=sharing
When available: With publication

Supporting Documents
Document types: None

Additional Information
Who can access the data: anyone
Types of analyses: anything
Mechanisms of data availability: without ivestigator support
